# Clinical Evaluation of a Polyethylene Glycol Derivative Rinse for Xerostomia

**DOI:** 10.3390/dj14030181

**Published:** 2026-03-18

**Authors:** Mabi L. Singh, Bryan Davis, Tiffany Bairos, Joseph Cimmino, Isha Singh, Minjung Ahn, Kwang Nho

**Affiliations:** 1 Division of Oral Medicine, Department of Diagnostic Sciences, Tufts University School of Dental Medicine, Boston, MA 02111, USA; mabi_l.singh@tufts.edu (M.L.S.); tiffany.bairos@tufts.edu (T.B.); joseph.cimmino@tufts.edu (J.C.); isha.singh@tufts.edu (I.S.); 2 Colorado Otolaryngology Associates, Colorado Springs, CO 80909, USA; bdavis@coloradoent.com; 3 SunBio, Inc., 129 Eunbong-ro, Namdong-gu, Incheon 21639, Republic of Korea; mjahn@sunbio.com

**Keywords:** xerostomia, polyethylene glycol (PEG) derivative, artificial saliva substitute, mouth rinses, clinical trial

## Abstract

**Background/Objectives**: This study aimed to evaluate the effect of a mouth rinse, MucoPEG™, containing a polyethylene glycol derivative, on oral dryness, compared to Biotène^®^ Dry Mouth Gentle Oral Rinse (Biotène^®^). **Methods**: Forty-two subjects with mild oral dryness with a Challacombe scale score of 1 or more were enrolled in the study across two sites using an open-label randomized crossover design. Subjects used either Biotène^®^ or MucoPEG™ twice daily for two weeks (Period 1) with a one-week washout period and then crossed over to the other product for two weeks (Period 2). The subjects provided a rating on a Visual Analogue Scale (VAS) for tongue and oral dryness and completed the Dry Mouth Relief Questionnaire (DMRQ), Dry Mouth Product Performance and Attributes Questionnaire (PPAQ), and Dry Mouth Inventory (DMI). **Results**: Both MucoPEG™ and Biotène^®^ demonstrated overall improvements in oral dryness symptoms with no statistically significant difference observed between products when both periods were combined. However, a statistically significant difference favoring MucoPEG™ was observed during Period 2. No significant sequence or period effects were detected. DMRQ and DMI responses were generally comparable between products. However, MucoPEG™ was associated with higher patient-reported ratings for sustained moisturizing and lubricating effects at 240 min post-application on the PPAQ only. No adverse events were reported. **Conclusions**: In this study MucoPEG™ demonstrated clinical performance comparable to that of Biotène^®^ in improving symptoms of oral dryness and was well tolerated. Although outcomes showed no significant differences between the two products, a subset of patient-reported outcomes suggests a potential advantage of MucoPEG™ in sustained symptom relief, consistent with its PEG-derivative formulation mechanism. However, these observations require validation. Further studies using parallel-group designs may help to clarify potential differences in long-term and sustained efficacy, thereby supporting the potential value of MucoPEG™ as an alternative therapeutic option for the management of xerostomia.

## 1. Introduction

Xerostomia, the subjective sensation of oral dryness [1], can occur with or without a reduction in saliva production [2,3,4] as there is no direct correlation between the volume of saliva produced and the perception of dryness [5]. This condition affects up to 50% of the population, making it a prevalent oral health issue [4]. Individuals experiencing oral dryness may often underestimate the severity of their symptoms [6]. The Challacombe Clinical Oral Dryness Score scale evaluates clinically observable signs of salivary hypofunction, and combining this with VAS scores allows for the assessment of both clinical signs and patient perception, providing a more comprehensive evaluation of treatment effects [7].

Xerostomia can be associated with various medical conditions such as mental health conditions, diabetes, allergies, and Sjögren’s disease. It is also a common side effect of numerous drugs such as anti-hypertensives, anti-psychotics, and decongestants [8,9,10]. Common symptoms associated with oral dryness include taste disturbances, halitosis, and mouth ulcers. Additionally, xerostomia can significantly impact a patient’s quality of life by impairing speech, chewing, and swallowing as well as causing a burning sensation [11,12,13].

Current management strategies for oral dryness include symptom-relieving measures such as topical saliva substitutes, oral moisturizers, saliva stimulants, and frequent sips of water. However, evidence supporting the effectiveness of any specific topical therapy is limited [2,3]. In addition, artificial saliva substitutes only offer temporary relief due to their rapid clearance from the oral cavity. Consequently, there remains a need for formulations capable of providing more sustained mucosal hydration.

Many additives to toothpastes, rinses, gels, and gums have been studied to assess their effectiveness in relieving symptoms of xerostomia and increasing saliva secretion. Such additives and elements include, but are not limited to, arginine, xylitol, olive oil, malic acid, and betaine [14,15]. While products like Biotène^®^ and Hydris™ have emerged as options for treating dry mouth in recent years, there is still growing demand for more effective and long-lasting solutions for dry mouth relief.

Polyethylene glycol (PEG) is a polyether compound known for its low toxicity and is utilized in various products [16,17,18] including drug delivery systems [19]. PEG exhibits a moisturizing effect when formulated in aqueous liquids due to its exceptional ability to retain water. Structurally, PEG is a polymer composed of repeating units of ethylene glycol, represented as -(CH_2_CH_2_O)_n_-, where each repeating unit has a molecular weight of 44 Da. Within this structure, each oxygen atom acts as a binding site for water molecules, in which hydrogen bonds form between the hydrogen atoms of water and the oxygen atoms of PEG. Research suggests that approximately two to three molecules of water bind to each oxygen atom in PEG [20]. Beyond simple hydration, PEG derivatives incorporating reactive functional groups may interact with mucosal surfaces, potentially enhancing retention within the oral cavity.

The primary ingredient in MucoPEG™ is a derivative of polyethylene glycol (PEG). General PEG is used as an excipient and additive in various medicinal, cosmetic, and food products. However, the PEG derivative used in MucoPEG™ is different from general PEG in that it has a functional group that reacts with amines in oral mucosa via amide bonds, while also exhibiting the hydrophilic characteristics of PEG. This formulation concept differs from conventional saliva substitutes that rely primarily on thickeners or lubricating excipients. The theoretical basis of this approach is to provide not only immediate symptomatic relief but also potentially more sustained mucosal hydration. SunBio, Inc. developed MucoPEG™ with the intention of alleviating dry mouth symptoms in individuals with xerostomia [21].

Previous studies have examined commercially available saliva substitutes and dry mouth products primarily in terms of symptom relief, patient preference, and perceived duration of moisturizing effects. While most formulations improve oral dryness, consistent evidence of clear superiority among products has been limited, highlighting the relevance of comparing products with differing formulation concepts.

This study aimed to evaluate the effectiveness of a polyethylene glycol (PEG) derivative-based oral rinse by comparing MucoPEG™ and Biotène^®^ in individuals experiencing oral dryness. In addition, patient-reported outcomes were examined, including product safety, perceived performance, changes in dry mouth symptoms, and mouth-feel qualities.

## 2. Materials and Methods

### 2.1. Materials

#### 2.1.1. Investigational Device

MucoPEG™ received FDA clearance on 5 November 2019, with the 510(k) number K190144. It is an artificial saliva product primarily composed of a polyethylene glycol (PEG) derivative, which is designed to form covalent bonds with oral epithelial cells. The additives in MucoPEG™ include mint as a flavoring agent and sodium bicarbonate as a buffering agent for pH maintenance.

Unlike other commercial products such as Hydris and Biotène^®^, MucoPEG™ does not contain thickeners (e.g., cellulose gum, xanthan gum, or carbomer), surfactants (e.g., Poloxamer), preservatives (e.g., cetylpyridinium chloride), colorants, or sweeteners (e.g., sorbitol, sodium saccharin, or sucralose).

MucoPEG™ is available on prescription for home use. A single dose of 1 g MucoPEG™ powder is dissolved in 20 mL (approximately 0.67 fluid oz) of water using the accessory bottle and shaken well for about 10 s for complete mixing. When completely mixed, the solution is gargled and swished inside the oral cavity for 30–60 s and then spat out.

The packets of MucoPEG™ should be stored in a dry environment and kept in the freezer at −4 ± 9 °F. MucoPEG™ is not sterile but conforms to ISO 10993-1 standards biological evaluation of medical devices, including tests for cytotoxicity, sensitization, and irritation [22].

Given the open-label design and at-home preparation of the powder formulation, participants received standardized instructions and training on product reconstitution at the initial visit. Participants were instructed to record product storage location, time of use, and missed doses in daily patient diaries. Participants returned their product containers and patient diaries at each study visit, and returned materials were reviewed to assess adherence.

#### 2.1.2. Comparator Information

Biotène^®^ Dry Mouth Gentle Oral Rinse [23] is specially formulated to clean and refresh the mouth, relieve dryness, and help soothe oral tissues. Biotène^®^ contains a combination of moisturizers and lubricants to provide immediate and long-lasting dry mouth symptom relief for up to 4 h, as measured in a 7-day clinical study. It is one of the leading over-the-counter products on the market. The manufacturers suggest gargling approximately 15 mL (one tablespoon) of Biotène^®^ for 30 s, and then spitting it out. Biotène^®^ can be used as needed up to 2–3 times a day [23].

### 2.2. Study Plan

#### 2.2.1. Study Purpose

The primary aim of this clinical trial was to assess and compare the effectiveness of MucoPEG™ and Biotène^®^ in subjects with oral dryness. Patient-reported outcomes regarding the safety and performance of the products, changes in dry mouth symptoms, and assessed mouth-feel qualities were also assessed.

#### 2.2.2. Study Design

The study employed an open-label randomized crossover design, in which patients were randomly assigned to receive either MucoPEG™ or Biotène^®^ during the first period. They were instructed to use the assigned oral rinse twice daily for two weeks. After a one-week wash-out period, patients switched to the alternate treatment. The study was reported in accordance with the CONSORT 2025 guidelines (Supplementary Table S1).

#### 2.2.3. Sample Size Calculation

The change in Visual Analogue Scale (VAS) score associated with each product during the first and second treatment periods was calculated. Since the VAS was constructed such that 0 = not dry and 10 = very dry, symptom improvement is represented by a negative change in its score. The hypotheses were to test whether patients would experience greater improvement in their symptoms after a course of MucoPEG™ treatment. Assuming an average difference between randomization groups of −1.25 with a common standard deviation of 1.50 and a Type I error of 0.05, an effective sample size of 38 patients provided 80% power to reject the null hypothesis of no difference between MucoPEG™ and Biotène^®^.

For comparing the percentage of patients reporting “Very Good” or “Significant/Excellent” responses in the Dry Mouth Relief Questionnaire (DMRQ), an effective sample size of 32 patients provided 80% power to demonstrate the superiority of MucoPEG™ over Biotène^®^, assuming a difference in percentages of 0.20 with a standard deviation of 0.45 and Type I error rate of 0.05.

Given that the effective sample size for the VAS scale analysis was greater than that for the DMRQ analysis, an a priori calculation was conducted and total enrollment of approximately 42 patients was aimed for, allowing for a 10% loss to follow-up.

#### 2.2.4. Randomization

Subjects were randomized in a 1:1 ratio to one of two treatment sequences (Group A or Group B) according to a crossover study design. Randomization determined the order in which subjects received the two study products and was performed prior to the initiation of treatment.

**Figure 1:**
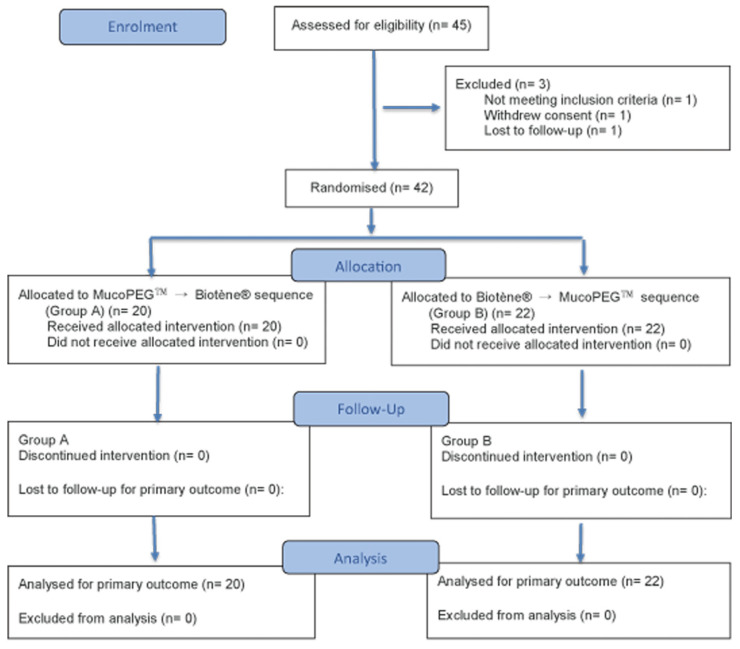
CONSORT flow diagram of the randomized crossover study. A total of 45 subjects were enrolled across two study sites, of whom 42 were randomized and included in the intention-to-treat (ITT) analysis. Subjects were randomly assigned in a 1:1 ratio to one of two treatment sequences: MucoPEG™ followed by Biotène^®^ or Biotène^®^ followed by MucoPEG™. Each treatment period lasted two weeks and was separated by a one-week washout period. Three subjects were excluded prior to randomization due to screen failure, withdrawal of consent, or loss to follow-up (n = 1 each). The final number of participants differed slightly between sequences (20 vs. 22), reflecting site-based enrollment targets and sequence randomization within each study site.

Subjects assigned to Group A received MucoPEG™ for two weeks during Period 1, followed by a one-week washout period, and then crossed over to Biotène^®^ for two weeks during Period 2. Subjects assigned to Group B followed the opposite sequence, receiving Biotène^®^ during Period 1 and MucoPEG™ during Period 2. Period 1 included Visits 2–4, and Period 2 included Visits 5–7, with the washout period occurring between the two treatment periods (Figure 1).

Randomization was performed using a 1:1 allocation scheme for treatment sequence; however, the final number of participants differed slightly between sequences (20 vs. 22) due to study conduct and analysis set definitions. This minor imbalance reflects site-based enrollment targets and sequence randomization within each study site, rather than a deviation from the intended randomization scheme.

Due to differences in product formulation, preparation, and packaging, participant blinding was not feasible. However, outcome assessments relied primarily on standardized patient-reported instruments, and statistical analyses were conducted by blinded statisticians to minimize potential assessment bias.

#### 2.2.5. Statistical Analysis of Clinical Data

Data were descriptively summarized as tables and/or graphs. Continuous variables, such as the patient’s assessment of mouth dryness using the VAS, were summarized as the mean, standard deviation, number of non-missing observations, median, minimum, and maximum by product used (MucoPEG™ or Biotène^®^) and by period (before or after washout). Discrete variables, including ordinal or binary responses to the Dry Mouth Relief Questionnaire, Product Performance and Attributes Questionnaire, etc., were summarized as frequency counts and percentages by product and period.

The intention-to-treat (ITT) analysis population includes all patients enrolled in the study, analyzed according to the group to which they were randomized. The per protocol analysis population is a subset of the ITT population and excludes patients with protocol deviations that could affect the measurement of the primary endpoint, such as the use of over-the-counter products for dry mouth during the study.

Hypothesis testing for the primary endpoints was conducted using data from the intention-to-treat population. The change in VAS associated with each product was calculated as the VAS value at the end of a treatment period minus the VAS value before the first dose in the same period. The difference in mean change in VAS for dry mouth between MucoPEG™ and Biotène^®^ was tested using Student’s *t*-test. Period-specific analyses were conducted to explore treatment effects within each study period.

Given the two-period crossover design, potential sequence effects were explored by comparing VAS changes between treatment sequences (Group A vs. Group B; MucoPEG™ → Biotène^®^ vs. Biotène^®^ → MucoPEG™) using two-sample *t*-tests. Period effects were evaluated using paired *t*-tests comparing VAS changes between Period 1 and Period 2 across subjects.

All statistical tests were two-sided with a significance level of 0.05. These additional analyses were conducted to assess the robustness of treatment comparisons within the crossover framework.

The difference in the proportion of patients rating the relief of dry mouth as “Very Good” or “Significant/Excellent” was tested using a binomial test of difference between two proportions. Statistical methods more suitable for smaller sample sizes, such as exact binomial tests or bootstrapping, were considered. Outcomes were analyzed by treatment and period in accordance with the crossover study design. No formal carryover adjustment model was implemented; however, sequence and period effects were explored to evaluate potential residual effects within the crossover design.

### 2.3. Methods

A total of forty-five subjects were prospectively enrolled across two study sites in the United States. Of these, forty-two subjects were randomized and included in the intention-to-treat (ITT) analysis, while three subjects were not randomized due to screen failure, withdrawal of consent, or loss to follow-up prior to randomization.

Twenty-two subjects were enrolled at site 1, of whom twenty-one completed the study. At site 2, twenty-three subjects were enrolled and twenty-one completed the study over approximately 42 days spanning from April to June 2022.

Subjects were recruited from the patient base through methods including posting flyers and word-of-mouth referrals at a dental school and private practice. As an inclusion criterion, subjects were required to have a score of 1 or higher on the Challacombe Clinical Oral Dryness Score scale.

#### 2.3.1. Visual Analog Scale (VAS)

The VAS was provided to the subjects, who were instructed to rate their oral and tongue dryness by placing a single vertical mark on a continuous line from 0 (“Not dry at all”) to 10 (“Very dry”). The study team used a 6-inch ruler with centimeter and millimeter markings to measure where the line fell, and that number was recorded.

#### 2.3.2. Dry Mouth Relief Questionnaire (DMRQ)

This questionnaire asked only one question: “Does the product relieve the discomfort of dry mouth?”. Subjects answered “None”, “Not enough”, “Some/Good”, “Very good”, or “Significant/Excellent”.

#### 2.3.3. Challacombe Scale of Clinical Oral Dryness

The Challacombe scale [7] works as an additive score of 1 to 10, with 1 being the least and 10 being the most severe for oral dryness. Score changes over time can be used to monitor symptom progression or regression. The patients’ scale scores were recorded on Visit 1 (screening) and Visit 2–7 (pre-use) by the Principal Investigator or designee.

#### 2.3.4. Dry Mouth Inventory (DMI) [3]

The subjects could choose to answer “Disagree”, “Agree a little”, “Agree”, or “Strongly agree” to the following statements.

1. My mouth feels dry.
2. I have difficulty eating dry foods.
3. I get up at night to drink.
4. My mouth feels dry when eating a meal.
5. I sip liquids to aid in swallowing food.
6. I suck sweets or cough lollies to relieve dry mouth.
7. My lips stick to the teeth.
8. My tongue sticks to the roof of my mouth.

#### 2.3.5. Product Performance and Attributes Questionnaire (PPAQ)

The PPAQ was completed by the patient at each time point (5, 30, 60, 120, and 240 min) after the product was administered. Subjects answered “Poor”, “Fair”, “Good”, “Very good”, or “Excellent”.

## 3. Results

### 3.1. Disposition of Subjects

A total of 42 subjects were randomized 1:1 into one of two treatment groups: 20 subjects were assigned to Group A (MucoPEG™ in Period 1 followed by Biotène^®^ in Period 2) and 22 subjects were assigned to Group B (Biotène^®^ in Period 1 followed by MucoPEG™ in Period 2). This is summarized in Figure 1.

### 3.2. Demographics (Intention-to-Treat)

Two sites at different geographic locations were used in this study. Subjects recruited at Site 1 and Site 2 exhibited mean age ranges of 51.3 ± 16.4 years with a median age of 54.0 years (Range: 23.0–75.0) and 68.6 ± 10.6 years with a median age of 67.0 years (Range: 50.0–93.0), respectively.

At Site 1, out of the 21 recruited subjects, 14 (66.7%) were under 62 years of age, while the remaining 7 (33.3%) were 62 years or older. Conversely, at Site 2, 6 (28.6%) subjects were under 62 years, and the remaining 15 (71.4%) were 62 years or older.

At Site 1, 15 out of the 21 subjects (71.4%) identified as white, 4 (19.0%) as Black or African American, and 1 (4.8%) as Asian. In contrast, all 21 subjects (100%) recruited from Site 2 identified as white. The subjects’ demographics are displayed in Table 1.

### 3.3. VAS for Oral Dryness

The VAS score (0 = “Not dry at all” and 10 = “Very dry”) before any dose was taken was 6.1 ± 2.7 for MucoPEG™ and 5.6 ± 2.5 for Biotène^®^. The mean change in the VAS score from baseline for both the intention-to-treat (ITT) and per protocol (PP) datasets was comparable between MucoPEG™ and Biotène^®^.

For MucoPEG™, the mean change in the VAS score for oral dryness across both periods combined was −1.1 ± 2.3, compared with −0.5 ± 2.4 for Biotène^®^. The between-treatment difference in mean change was −0.59 points. Although the numerical reduction was greater with MucoPEG™, the difference between treatments did not reach statistical significance in the primary combined-period analysis (*p* = 0.13).

In period-specific analyses, the mean change during Period 1 was −0.8 ± 2.0 for MucoPEG™ and −0.7 ± 3.0 for Biotène^®^, with no statistically significant difference observed between treatments. During Period 2, the mean change was −1.5 ± 2.4 for MucoPEG™ and −0.4 ± 1.6 for Biotène^®^, and the between-treatment difference reached statistical significance (*p* = 0.05). Please refer to Table 2 for detailed data.

For both oral and tongue VAS outcomes, comparisons of VAS changes between treatment sequences (Group A vs. Group B) did not reveal statistically significant sequence effects (oral: *p* = 0.34; tongue: *p* = 0.35). Similarly, paired comparisons of VAS changes between Period 1 and Period 2 did not demonstrate statistically significant period effects (oral: *p* = 0.55; tongue: *p* = 0.54).

### 3.4. VAS for Tongue Dryness

The VAS questionnaire (0 = not dry, 10 = very dry) was utilized to assess the dryness of the patients’ tongues. Table 3 below provides a summary of tongue dryness scores across both periods combined. The mean combined-period change was −1.0 ± 2.4 for MucoPEG™ and −0.3 ± 2.6 for Biotène^®^. The between-treatment difference in mean change was −0.67 points. Although the numerical reduction was greater with MucoPEG™, the difference between treatments did not reach statistical significance in the combined-period analysis (*p* = 0.11).

In Period 1, no statistically significant difference was observed between products. In Period 2, a statistically significant difference favoring MucoPEG™ was observed (*p* = 0.03). Sequence and period analyses for tongue VAS did not identify statistically significant effects (sequence: *p* = 0.35; period: *p* = 0.54).

### 3.5. DMRQ Responses

The DMRQ consisted of one question (“Does the product relieve the discomfort of dry mouth?”), which the patients answered 2 h after product use at all visits. The proportions of patients with a favorable response at the last visit after 2 weeks of use, according to product and study site, are summarized in Table 4. Overall, the proportion of patients with a favorable response was comparable between Biotène^®^ (26.2%, 11/42) and MucoPEG™ (23.8%, 10/42) on the last visit (*p* = 0.6). In period-specific subgroup analyses, favorable response rates varied numerically between products. During Period 1, favorable responses were numerically higher for MucoPEG™ (25.0% vs. 13.6%), whereas in Period 2, higher rates were observed for Biotène^®^ (40.0% vs. 22.7%). By study site, responses were numerically higher for Biotène^®^ at Site 1 (38.1% vs. 23.8%) and for MucoPEG™ at Site 2 (23.8% vs. 14.3%). None of these subgroup differences reached statistical significance. These subgroup findings are descriptive in nature and should be interpreted cautiously.

### 3.6. Challacombe Scale of Clinical Oral Dryness

A score on the Challacombe scale of clinical oral dryness of 1 or more was used as the criterion for enrolling the subjects. The patients’ Challacombe scale scores were recorded by the principal investigator or designee at Visit 1 (screening) and Visits 2–7 (pre-use). Visits 2–4 corresponded to Period 1, and Visits 5–7 corresponded to Period 2. The results by study site, product, and visit are displayed in Table 5 below.

At Screening (Visit 1), the mean Challacombe scale score for both study sites combined was 4.2 ± 1.7, with a median score of 4.0 (Range: 1.0, 9.0). Subjects recruited at Site 1 and Site 2 had mean baseline scores of 3.5 ± 1.4 and 4.9 ± 1.7, respectively. In Period 1, the mean Challacombe scale score decreased from 3.7 ± 2.3 before MucoPEG™ use to 2.7 ± 2.1 after 2 weeks of treatment. For Biotène^®^, the score decreased from 4.0 ± 1.5 to 3.5 ± 1.1. Following the 1-week washout, in Period 2, the mean score for MucoPEG™ decreased from 3.9 ± 1.8 to 3.4 ± 1.2, and for Biotène^®^, from 3.0 ± 1.9 to 2.8 ± 1.4.

Reductions in Challacombe scale scores were observed after 2 weeks of use with both products. No statistically significant differences between treatments were identified in combined-period or period-specific analyses. These investigator-assessed findings were consistent with the patient-reported VAS outcomes.

### 3.7. Dry Mouth Inventory

The mean and median values of the responses for both periods, combined and categorized by site and product, are displayed in Table 6. For both sites combined, the mean and median total scores were identical. For MucoPEG™, the mean total score was 9.4 ± 5.3, with a median total score of 10.0 (Range: 0.0, 21.0). Similarly, for Biotène^®^, the mean total score was 9.4 ± 5.6, with a median total score of 10.0 (Range: 0.0, 21.0).

### 3.8. Product Performance and Attributes Questionnaire (PPAQ)

**Figure 2:**
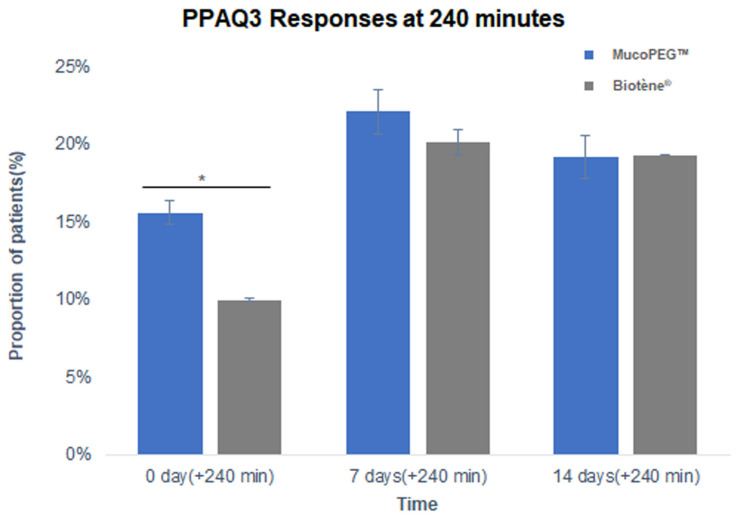
Proportion of patients giving a rating of 4–5 (“very good” or “excellent”) for the PPAQ3 questionnaire at 240 min after product use. The PPAQ3 consists of three questions: Q12 (regarding long-lasting dry mouth relief), Q13 (regarding long-lasting lubricating effect), and Q14 (regarding long-lasting moisturizing effect). Bars represent the mean proportion of patients giving scores of ≥4 across the three questions. The *p*-value at 0 days (+240 min) is 0.017 * (<0.05), and the *p*-values at 7 and 14 days (+240 min) are 0.262 and 1.

For the PPAQ, the patients answered several questions at different time points during each visit to determine the effectiveness and duration of effect of the two products. PPAQ1 and 2 are questionnaires about the efficacy of the product 5 and 30 min after using it, and PPAQ3 and 4 are questionnaires about the sustainability of the effect at 60, 120, and 240 min and the following morning. For the responses to most of the questions, there were no significant differences between the treatments. Figure 2 summarizes the subjects’ responses to the PPAQ3 questionnaire at 240 min, at which time point it was indicated that the patients preferred MucoPEG™ as it provided longer-lasting dry mouth relief, lubrication, and moisturization.

## 4. Discussion

Saliva plays a crucial role in oral health, serving various functions such as lubrication, protection against pathogens, and facilitation of digestion. However, salivary hypofunction can arise from a range of causes, both reversible and irreversible. Irreversible causes include autoimmune diseases such as Sjögren’s disease, diabetes, and certain medical treatments such as radiation therapy. Reversible causes may include medications, dehydration, mental illness, and environmental factors such as dry air [9,24,25,26,27,28,29,30]. Given that hyposalivation and xerostomia can have multiple causes, variable severity, a range of symptoms, and a subjective component, finding effective treatments is complex and patient-specific. Patients often try many products to find relief. A 2012 study compared two rinses, both containing an aqueous solution of xylitol, sodium fluoride, cetylpyridinium chloride, sodium chloride, and spearmint flavoring. In addition to these ingredients, rinse 2 also contained propylene glycol, aloe vera, glycerin, and citric acid. Both rinses were found to be effective in relieving some symptoms of dry mouth in xerostomic patients taking three or more medications, but neither were found to relieve symptoms in xerostomic patients taking two or fewer medications [31].

Many widely utilized oral products incorporate a highly hydrophilic, petroleum-based polyethylene glycol (commonly referred to as PEG, or PEG-based formulations) as an excipient or surfactant. PEG in general is well-known for its moisturizing effect when formulated as an aqueous liquid, due to its excellent ability to retain water. These formulations rapidly hydrate and, upon contact with mucosal surfaces, establish a layer, which, in essence, is vital for effective mucosal adhesion. Thus, PEG acts as a humectant, attracting and retaining moisture. The PEG derivative in MucoPEG™, which has an added functional group, allows the PEG to bind to dry oral mucosa, providing continuous relief for dry mouth discomfort.

MucoPEG™, based on PEG derivatives, offers prolonged relief from dry mouth discomfort compared to traditional PEG-based formulations. By immobilizing PEG on oral epithelial cells and tissues, MucoPEG™ mimics the natural salivary film, providing sustained relief from mucosal dryness. The observed preference of the patients after 240 min is consistent with this mechanism and highlights the potential functional importance of surface attachment by PEG derivatives in the management of xerostomia. Importantly, the safety of PEG usage is well-established.

Polymeric oral products can play a vital role in xerostomia management [32] and improving the quality of life of individuals affected by salivary hypofunction and resultant oral dryness. This contribution is especially notable when combined with lifestyle changes, adjustments to medications that induce xerostomia, or the mitigation of other factors that contribute to dry mouth.

The demographic characteristics of the participants differed notably between Site 1 and Site 2. Specifically, the Site 1 participants exhibited a lower mean age compared to those at Site 2. In contrast, the Site 2 population had an older average age and a higher prevalence of clinically significant medical histories, including a higher rate of cancer cases. Additionally, the racial demographics varied between the sites, with Site 1 having a more diverse participant population compared to Site 2. These inter-site demographic differences highlight the importance of considering site-specific factors when interpreting the study’s results and drawing generalized conclusions. The Poolability Analysis, which was conducted in the intention-to-treat (ITT) population and closely resembled the per protocol (PP) analysis (the data not presented).

Subjects using MucoPEG™ exhibited numerically greater reduction in VAS scores compared to those using Biotène^®^, indicating an improvement in symptoms of oral dryness. However, this improvement did not reach statistical significance, and thus, the null hypothesis was not rejected. In period-specific analyses, statistically significant differences favoring MucoPEG™ were observed during Period 2 for both oral and tongue dryness. Nevertheless, no statistically significant sequence or period effects were identified, suggesting that treatment order and study period did not materially influence outcomes. These findings should therefore be interpreted cautiously. There was a site-specific improvement in the subjects using MucoPEG™ compared to those using Biotène^®^, although this was not statistically significant, possibly due to differences in age, health status, and severity of oral dryness between the participants of different study sites. Subjects with more severe oral dryness symptoms at baseline may have perceived greater relief from MucoPEG™, potentially by reducing intraoral friction and enhancing lubrication between oral surfaces through improved pellicle formation [33].

Products are constantly being developed [34] to treat the subjective sensation of oral dryness, including chewing gum, mucoadhesive tablets, oil pulling, gene therapy, electrostimulation, medical management, moisturizing sprays, saliva substitutes with methylcellulose, and oily emulsions [35,36,37,38,39,40,41,42,43]. However, the effectiveness of these products varies, with factors such as oral clearance influencing the duration of their effect. There was no strong evidence from a review of thirty-six randomized controlled trials proving that any topical therapy is effective for relieving the symptom of dry mouth [4]. Notably, some oral rinses, particularly those containing antiseptic compounds, may worsen symptoms in individuals experiencing oral dryness. Jose et al. compared various mouthwashes and found that those containing humectants (e.g., glycerin, sorbitol, and xylitol) were more effective in trapping and retaining moisture for an extended period compared to water alone [6]. In another study, Varghese et al. [44] concluded that individuals experiencing xerostomia perceived moisturizing mouthwashes as gentle, moisturizing, soothing, and refreshing, with many expressing an intention to continue using them. In this study, MucoPEG™, formulated with PEG derivatives, demonstrated comparable clinical performance to Biotène^®^ across outcome measures. It is noteworthy that no adverse events were reported with either of the products, and the safety of the versatile polymer polyethylene glycol (PEG) is well-established [45].

On the PPAQ, MucoPEG™ was associated with higher ratings for sustained moisturizing and lubricating effects at the 240-min time point at baseline. Although these differences were not consistently observed at later visits, the findings may be consistent with the hydrophilic properties of PEG derivatives. Mechanistically, MucoPEG™ coats the oral mucosa with PEG derivatives. It is hypothesized that repeated use may increase mucosal coverage. Accordingly, the moisturizing effect is sustained. However, patients may become less sensitive to the effect over time. Further investigation in appropriately powered parallel-group studies would be required to confirm differences in duration of symptom relief.

The findings from the post-product use questionnaire, Dry Mouth Inventory, and DMRQ revealed no significant differences between the two products compared. Investigator-assessed Challacombe scores decreased with both products, and no statistically significant between-treatment differences were identified. The consistency between investigator-assessed and patient-reported outcomes supports the robustness of the findings within the constraints of the open-label crossover design.

This study had several limitations that should be acknowledged. First, the participants needed to have only mild symptoms of oral dryness to be eligible, as reflected by the low Challacombe score for qualification. This may limit the generalizability of the findings to individuals with moderate to severe symptoms. Second, the study employed an open-label crossover design, which may have introduced perception-related bias due to the lack of participant blinding. Blinding was not feasible because of the preparation procedure required for MucoPEG™, which made concealment of product identity impractical. Although subjective outcome measures may be susceptible to expectation effects, sequence and period analyses did not demonstrate statistically significant effects, suggesting that treatment order did not materially influence the observed outcomes. Lastly, the sample size was modest and the study was not powered to detect small between-treatment differences.

Overall, MucoPEG™ demonstrated clinical performance comparable to that of Biotène^®^ in improving symptoms of oral dryness and was well tolerated. Period-specific and patient-reported findings suggest a potential advantage in sustained symptom relief; however, confirmation in larger, parallel-group randomized studies is warranted. Future investigations incorporating objective assessments, such as mucosal wetness and salivary fluid film thickness measured at multiple time points, may provide complementary evidence alongside patient-reported outcomes. Inclusion of participants with moderate to severe levels of oral dryness may further clarify treatment responsiveness across varying symptom severity.

## 5. Conclusions

In conclusion, both MucoPEG™ and Biotène^®^ were safe and effective in improving symptoms of oral dryness. Clinical performance was generally comparable between the two products in the combined-period analysis. Period-specific and patient-reported findings suggest a potential advantage of MucoPEG™ in sustained symptom relief; however, these observations should be interpreted cautiously. These characteristics suggest that MucoPEG™ may represent an alternative therapeutic option in the management of xerostomia, with potential advantages related to sustained symptom relief. Further investigation in larger, randomized, parallel-group studies may help to confirm these observations.

## Figures and Tables

**Table 1:** Participants’ demographics (ITT) by study site.

Data	Site 1	Site 2	Both Sites Combined
Age at consent (years)	51.3 ± 16.4 (21)	68.6 ± 10.6 (21)	60.0 ± 16.2 (42)
	54.0 [23.0, 75.0]	67.0 [50.0, 93.0]	62.5 [23.0, 93.0]
Age group			
Under 62 years	66.70%	28.60%	47.60%
Over 62 years	33.30%	71.40%	52.40%
Sex			
Female	57.10%	38.10%	47.6% (20/42)
Male	42.90%	61.90%	52.4% (22/42)
Race			
American Indian or Alaska Native	0.0% (0/21)	0.0% (0/21)	0.0% (0/42)
Asian	4.8% (1/21)	0.0% (0/21)	2.4% (1/42)
Black or African American	19.0% (4/21)	0.0% (0/21)	9.5% (4/42)
Native Hawaiian or Pacific Islander	0.0% (0/21)	0.0% (0/21)	0.0% (0/42)
White	71.4% (15/21)	100.0% (21/21)	85.7% (36/42)
Not Reported	4.8% (1/21)	0.0% (0/21)	2.4% (1/42)
Ethnicity			
Not Hispanic or Latino	85.7% (18/21)	100% (21/21)	92.9% (39/42)
Hispanic or Latino	14.3% (3/21)	0.0% (0/21)	7.1% (3/42)
Pregnancy Status			
Patient is of non-childbearing status	50.0% (6/12)	87.5% (7/8)	65.0% (13/20)
Patient verbally confirmed they are not pregnant	50.0% (6/12)	12.5% (1/8)	35.0% (7/20)

For continuous variables, the estimates are presented as the mean ± standard deviation (number of non-missing observations) and median [minimum, maximum]. For categorical variables, the estimates are displayed as percentages (number of responses/number of patients at-risk).

**Table 2:** Change in VAS scores for oral dryness (ITT, primary endpoint, n = 42).

Change in VAS	MucoPEG™			Biotène^®^			Mean Difference: MucoPEG™–Biotène^®^ (Upper Limit of 90% Confidence Interval)	*p*-Value
	VAS Before Any Dose on First Day	VAS After Last Dose on Final Day	Change in VAS	VAS Before Any Dose on First Day	VAS After Last Dose on Final Day	Change in VAS		
Both periods combined	6.1 ± 2.7 (42) 6.8 [0.2, 10.0]	5.0 ± 2.7 (42) 5.6 [0.1, 9.6]	−1.1 ± 2.3 (42) −1.0 [−6.8, 5.9]	5.6 ± 2.5 (42) 5.8 [0.3, 9.2]	5.0 ± 2.7 (42) 4.8 [0.1, 9.9]	−0.5 ± 2.4 (42) −0.2 [−5.7, 6.1]	−0.59 (0.26)	0.13
Period 1	4.9 ± 3.2 (20) 5.0 [0.2, 10.0]	4.1 ± 2.8 (20) 4.0 [0.2, 9.6]	−0.8 ± 2.0 (20) −0.6 [−3.5, 5.9]	6.5 ± 2.0 (22) 7.3 [1.6, 8.8]	5.9 ± 2.3 (22) 5.9 [2.1, 9.9]	−0.7 ± 3.0 (22) −0.6 [−5.7, 6.1]	−0.10 (1.24)	0.45
Period 2	7.2 ± 1.5 (22) 7.4 [3.1, 10.0]	5.7 ± 2.4 (22) 6.8 [0.1, 8.8]	−1.5 ± 2.4 (22) −1.1 [−6.8, 2.9]	4.5 ± 2.7 (20) 4.7 [0.3, 9.2]	4.1 ± 2.8 (20) 4.2 [0.1, 9.3]	−0.4 ± 1.6 (20) −0.2 [−3.4, 2.8]	−1.06 (−0.00)	0.05

For continuous variables, the estimates are presented as the mean ± standard deviation (number of non-missing observations) and median [minimum, maximum]. Satterthwaite results were used for the *t*-test statistics.

**Table 3:** Change in VAS scores for tongue dryness (ITT, primary endpoint, n = 42).

Change in VAS	MucoPEG™			Biotène^®^			Mean Difference: MucoPEG™–Biotène^®^ (Upper Limit of 90% Confidence Interval)	*p*-Value
	VAS Before Any Dose on First Day	VAS After Last Dose on Final Day	Change in VAS	VAS Before Any Dose on First Day	VAS After Last Dose on Final Day	Change in VAS		
Both periods combined	5.8 ± 2.9 (42) 6.6 [0.2, 10.0]	4.8 ± 2.8 (42) 5.0 [0.2, 9.3]	−1.0 ± 2.4 (42) −0.7 [−5.7, 5.8]	5.2 ± 2.6 (42) 4.9 [0.3, 9.4]	4.8 ± 2.7 (42) 4.6 [0.2, 10.0]	−0.3 ± 2.6 (42) −0.2 [−5.9, 7.4]	−0.67 (0.23)	0.11
Period 1	4.6 ± 3.4 (20) 4.0 [0.2, 10.0]	4.0 ± 2.9 (20) 3.8 [0.3, 9.3]	−0.6 ± 2.4 (20) −0.4 [−4.7, 5.8]	6.2 ± 2.2 (22) 6.4 [1.3, 8.8]	5.7 ± 2.4 (22) 5.7 [2.0, 10.0]	−0.4 ± 3.3 (22) −1.0 [−5.9, 7.4]	−0.16 (1.36)	0.43
Period 2	6.9 ± 1.8 (22) 7.0 [3.2, 10.0]	5.6 ± 2.5 (22) 6.3 [0.2, 8.9]	−1.4 ± 2.4 (22) −1.0 [−5.7, 3.0]	4.1 ± 2.7 (20) 4.0 [0.3, 9.4]	3.9 ± 2.7 (20) 3.9 [0.2, 9.4]	−0.2 ± 1.3 (20) −0.0 [−3.2, 1.9]	−1.16 (−0.18)	0.03

For continuous variables, the estimates are presented as the mean ± standard deviation (number of non-missing observations) and median [minimum, maximum]. Satterthwaite results were used for the *t*-test statistics.

**Table 4:** Differences in DMRQ responses by period and study site subgroups (ITT, primary endpoints).

Proportion of Patients with a Favorable Response at Last Visit	MucoPEG™	Biotène^®^	Proportion Difference: MucoPEG™–Biotène^®^ (Lower Limit of 90% Confidence Interval)	*p*-Value *
Overall	23.8% (10/42)	26.2% (11/42)	−2.4 (−17.9)	0.6
Period 1	25.0% (5/20)	13.6% (3/22)	11.4 (−8.6)	0.17
Period 2	22.7% (5/22)	40.0% (8/20)	−17.3 (−40.5)	0.89
Site 1	23.8% (5/21)	38.1% (8/21)	−14.3 (−37.5)	0.84
Site 2	23.8% (5/21)	14.3% (3/21)	9.5 (−10.3)	0.21

A favorable response in the Dry Mouth Relief Questionnaire (DMRQ) is “4—very good” or “5—significant/excellent”. For categorical variables, the estimates are displayed as percentages (number of patients with a favorable response at last visit/the number of patients eligible for taking the Dry Mouth Relief Questionnaire). * *p*-values for test of difference between two proportions.

**Table 5:** Summary of Challacombe scale scores from oral tissue examination (ITT), by both study sites combined, product and visit.

Data Item	Visit	Site 1			Site 2			All Study Sites Combined		
		Screening	MucoPEG™	Biotene^®^	Screening	MucoPEG™	Biotene^®^	Screening	MucoPEG™	Biotene^®^
Total score	1	3.5 ± 1.4 (21) 4.0 [1.0, 6.0]			4.9 ± 1.7 (21) 5.0 [2.0, 9.0]			4.2 ± 1.7 (42) 4.0 [1.0, 9.0]		
	2 (Pre)		2.8 ± 2.0 (11) 2.0 [1.0, 8.0]	3.4 ± 1.5 (10) 3.0 [2.0, 6.0]		4.7 ± 2.2 (9) 4.0 [2.0, 8.0]	4.5 ± 1.4 (12) 4.5 [2.0, 7.0]		3.7 ± 2.3 (20) 3.5 [1.0, 8.0]	4.0 ± 1.5 (22) 4.0 [2.0, 7.0]
	3 (1 wk)		2.3 ± 1.3 (11) 2.0 [1.0, 5.0]	3.4 ± 1.3 (10) 3.0 [2.0, 6.0]		4.4 ± 1.7 (9) 4.0 [2.0, 8.0]	3.7 ± 1.8 (12) 4.0 [1.0, 7.0]		3.3 ± 1.8 (20) 3.0 [1.0, 8.0]	3.5 ± 1.6 (22) 3.5 [1.0, 7.0]
	4 (2 wk)		1.9 ± 1.7 (11) 2.0 [0.0, 5.0]	3.0 ± 1.2 (10) 3.0 [1.0, 5.0]		3.7 ± 2.1 (9) 3.0 [1.0, 7.0]	3.8 ± 0.9 (12) 4.0 [2.0, 5.0]		2.7 ± 2.1 (20) 2.0 [0.0, 7.0]	3.5 ± 1.1 (22) 4.0 [1.0, 5.0]
	Washout Period/1 wk									
	5 (Pre)		3.4 ± 1.8 (10) 3.0 [1.0, 7.0]	2.3 ± 1.2 (11) 2.0 [1.0, 4.0]		4.3 ± 1.7 (12) 4.0 [1.0, 8.0]	3.9 ± 2.2 (9) 4.0 [1.0, 7.0]		3.9 ± 1.8 (22) 4.0 [1.0, 8.0]	3.0 ± 1.9 (20) 3.0 [1.0, 7.0]
	6 (1 wk)		2.7 ± 1.3 (10) 3.0 [1.0, 4.0]	2.8 ± 1.3 (11) 2.0 [2.0, 6.0]		3.5 ± 1.7 (12) 4.0 [1.0, 6.0]	4.3 ± 1.5 (9) 4.0 [3.0, 8.0]		3.1 ± 1.6 (22) 3.5 [1.0, 6.0]	3.5 ± 1.5 (20) 3.0 [2.0, 8.0]
	7 (2 wk)		2.7 ± 0.8 (10) 2.5 [2.0, 4.0]	2.1 ± 1.3 (11) 2.0 [0.0, 5.0]		3.9 ± 1.2 (12) 4.0 [2.0, 6.0]	3.6 ± 1.2 (9) 4.0 [2.0, 6.0]		3.4 ± 1.2 (22) 3.0 [2.0, 6.0]	2.8 ± 1.4 (20) 2.5 [0.0, 6.0]

For continuous variables, the estimates are presented as the mean ± standard deviation (number of non-missing observations) and median [minimum, maximum].

**Table 6:** Summary of responses to Dry Mouth Inventory, both periods combined, by study site and product.

Data Item	Site 1		Site 2		All Study Sites Combined	
	MucoPEG™	Biotène^®^	MucoPEG™	Biotène^®^ *	MucoPEG™	Biotène^®^
My mouth feels dry	1.4 ± 0.9 (63) 1.0 [0.0,3.0]	1.3 ± 1.0 (63) 1.0 [0.0,3.0]	1.9 ± 1.0 (63) 2.0 [0.0,3.0]	1.8 ± 0.9 (62) 2.0 [0.0,3.0]	1.6 ± 1.0 (126) 2.0 [0.0,3.0]	1.5 ± 0.9 (125) 2.0 [0.0,3.0]
I have difficulty in eating dry foods	0.9 ± 1.0 (63) 1.0 [0.0,3.0]	0.9 ± 0.9 (63) 1.0 [0.0,3.0]	1.6 ± 1.1 (63) 2.0 [0.0,3.0]	1.6 ± 1.1 (62) 2.0 [0.0,3.0]	1.3 ± 1.1 (126) 1.0 [0.0,3.0]	1.3 ± 1.0 (125) 1.0 [0.0,3.0]
I get up at night to drink	0.9 ± 0.8 (63) 1.0 [0.0,3.0]	0.8 ± 0.8 (63) 1.0 [0.0,3.0]	1.7 ± 1.0 (63) 2.0 [0.0,3.0]	1.6 ± 1.0 (62) 2.0 [0.0,3.0]	1.3 ± 1.0 (126) 1.0 [0.0,3.0]	1.2 ± 1.0 (125) 1.0 [0.0,3.0]
My mouth feels dry when eating a meal	0.8 ± 0.8 (63) 1.0 [0.0,3.0]	1.0 ± 0.9 (63) 1.0 [0.0,3.0]	1.5 ± 0.9 (63) 2.0 [0.0,3.0]	1.5 ± 1.0 (62) 2.0 [0.0,3.0]	1.2 ± 0.9 (126) 1.0 [0.0,3.0]	1.2 ± 1.0 (125) 1.0 [0.0,3.0]
I sip liquids to aid in swallowing food	1.0 ± 0.9 (63) 1.0 [0.0,3.0]	1.1 ± 0.9 (63) 1.0 [0.0,3.0]	1.8 ± 1.1 (63) 2.0 [0.0,3.0]	1.7 ± 1.1 (62) 2.0 [0.0,3.0]	1.4 ± 1.1 (126) 1.0 [0.0,3.0]	1.4 ± 1.1 (125) 1.0 [0.0,3.0]
I suck sweets or cough lollies to relieve dry mouth	0.6 ± 0.8 (63) 0.0 [0.0,3.0]	0.6 ± 0.9 (63) 0.0 [0.0,3.0]	1.4 ± 1.1 (63) 2.0 [0.0,3.0]	1.5 ± 1.2 (62) 1.0 [0.0,3.0]	1.0 ± 1.1 (126) 1.0 [0.0,3.0]	1.0 ± 1.2 (125) 1.0 [0.0,3.0]
My lips stick to the teeth	0.8 ± 0.8 (63) 1.0 [0.0,3.0]	0.7 ± 0.8 (63) 0.0 [0.0,2.0]	0.8 ± 0.7 (63) 1.0 [0.0,2.0]	1.0 ± 0.9 (62) 1.0 [0.0,3.0]	0.8 ± 0.8 (126) 1.0 [0.0,3.0]	0.9 ± 0.9 (125) 1.0 [0.0,3.0]
My tongue stick to the roof of the mouth	0.8 ± 0.8 (63) 1.0 [0.0,3.0]	0.8 ± 0.9 (63) 1.0 [0.0,3.0]	0.9 ± 0.9 (63) 1.0 [0.0,3.0]	0.8 ± 0.9 (62) 1.0 [0.0,3.0]	0.8 ± 0.9 (126) 1.0 [0.0,3.0]	0.8 ± 0.9 (125) 1.0 [0.0,3.0]
Total score	7.2 ± 4.9 (63) 7.0 [0.0,19.0]	7.3 ± 5.3 (63) 7.0 [0.0,21.0]	11.6 ± 4.7 (63) 11.0 [1.0,21.0]	11.5 ± 5.2 (62) 12.0 [2.0,21.0]	9.4 ± 5.3 (126) 10.0 [0.0,21.0]	9.4 ± 5.6 (125) 10.0 [0.0,21.0]

For continuous variables, the estimates are presented as the mean ± standard deviation (number of non-missing observations) and median [minimum, maximum]. * Patient 102-006 had missing data for Visit 6 during Period 2; therefore, the number of responses was 62.

## Data Availability

The data presented in this study are available on request from the corresponding author due to privacy reasons.

## Supplementary Materials

The following supporting information can be downloaded at: https://www.mdpi.com/article/10.3390/dj14030181/s1. Supplementary Table S1: CONSORT 2025 checklist.
