# Supplementary material for: Clinical Evaluation of a Polyethylene Glycol Derivative Rinse for Xerostomia
Source: Dent J (Basel). 2026 Mar 18;14(3):181. doi: 10.3390/dj14030181 (PMC13024883; doi:10.3390/dj14030181)
Supplement: Supplementary file 1 [file dentistry-14-00181-s001.zip › dentistry-4077595-supplementary.pdf]

Supplementary Table S1. CONSORT 2025 Checklist for Reporting Randomized Trials

Section/Topic	Item No.	Checklist Item	Reported on Page No.
Title and Abstract	1a	Identification as a randomized trial in the title	Title page
Title and Abstract	1b	Structured summary of trial design, methods, results, and conclusions	Abstract
Introduction	2a	Scientific background and explanation of rationale	Introduction
Introduction	2b	Specific objectives or hypotheses	Introduction
Methods	3a	Description of trial design (randomized crossover trial) including allocation ratio	Methods – Study Design section
Methods	3b	Important changes to methods after trial commencement	Not applicable
Participants	4a	Eligibility criteria for participants	Methods – Participants
Participants	4b	Settings and locations where the data were collected	Methods – Study Sites

Interventions	5	Detailed description of the interventions for each group	Methods – Interventions
Outcomes	6a	Completely defined pre-specified primary and secondary outcome measures	Methods – Outcomes
Outcomes	6b	Any changes to trial outcomes after trial commencement	Not applicable
Sample Size	7a	How sample size was determined	Methods – Statistical Analysis section
Sample Size	7b	Interim analyses and stopping guidelines	Not applicable
Randomization – Sequence Generation	8a	Method used to generate the random allocation sequence	Methods – Randomization
Randomization – Sequence Generation	8b	Type of randomization and details of any restriction	Methods – Randomization
Allocation Concealment Mechanism	9	Mechanism used to implement the random allocation sequence	Methods – Randomization
Implementation	10	Who generated the allocation sequence, enrolled participants, and assigned participants to interventions	Methods – Randomization

Blinding	11a	Whether participants, personnel, and outcome assessors were blinded	Methods – Study Design
Blinding	11b	Description of similarity of interventions	Methods – Interventions
Statistical Methods	12a	Statistical methods used to compare groups for primary outcome	Methods – Statistical Analysis
Statistical Methods	12b	Methods for additional analyses including subgroup analyses	Methods – Statistical Analysis
Results – Participant Flow	13a	Number of participants randomized, received intended treatment, and analyzed for the primary outcome	Figure 1
Results – Participant Flow	13b	Losses and exclusions after randomization	Figure 1
Recruitment	14a	Dates defining recruitment and follow-up periods	Methods – Study Timeline
Recruitment	14b	Why the trial ended or was stopped	Not applicable
Baseline Data	15	Baseline demographic and clinical characteristics	Results – Baseline Characteristics
Numbers Analyzed	16	Number of participants	Results

		included in each analysis	
Outcomes and Estimation	17a	Results for each primary and secondary outcome	Results
Outcomes and Estimation	17b	Estimated effect size and precision	Results
Ancillary Analyses	18	Results of subgroup and adjusted analyses	Results
Harms	19	All important harms or unintended effects	Results – Safety
Discussion – Limitations	20	Trial limitations addressing sources of bias or imprecision	Discussion
Generalizability	21	Generalizability of the trial findings	Discussion
Interpretation	22	Interpretation consistent with results and limitations	Discussion
Registration	23	Registration number and name of trial registry	ClinicalTrials.gov NCT04986501
Protocol	24	Where the full trial protocol can be accessed	Not publicly available
Funding	25	Sources of funding and role of funders	This study was funded by SunBio, Inc.
